# A Low-Powered and Highly Selective Trap for Male *Aedes* (Diptera: Culicidae) Surveillance: The Male *Aedes* Sound Trap

**DOI:** 10.1093/jme/tjaa151

**Published:** 2020-08-02

**Authors:** Kyran M Staunton, Jacob E Crawford, Jianyi Liu, Michael Townsend, Yu Han, Mark Desnoyer, Paul Howell, Wei Xiang, Thomas R Burkot, Nigel Snoad, Scott A Ritchie

**Affiliations:** 1 College of Public Health, Medical and Veterinary Sciences, James Cook University, Smithfield, QLD, Australia; 2 Australian Institute of Tropical Health and Medicine, James Cook University, Smithfield, QLD, Australia; 3 Verily Life Sciences, South San Francisco, CA; 4 College of Science & Engineering, James Cook University, Smithfield, QLD, Australia

**Keywords:** *Aedes aegypti*, mosquito trap, dengue, male, sound lure

## Abstract

As *Aedes aegypti* (Linnaeus, Diptera: Culicidae) expands its global distribution and vectors a range of debilitating arboviruses there is an increased need for enhanced mosquito surveillance. Consequently, we developed a Male *Aedes* Sound Trap (MAST) that requires minimal power and is highly species-specific. Two different versions of the MAST were developed, one that uses synthetic pyrethroid to kill captured mosquitoes (MAST Spray) and another which has an internal divider to create a killing chamber in which a sticky panel can be placed to capture mosquitoes (MAST Sticky). We compared weekly capture rates of male *Ae. aegypti* and bycatch from the two MAST versions to those from BG-Sentinel (BGS) traps and Sound-producing BG-Gravid *Aedes* Traps (SGATs) throughout Cairns, northern Australia. Weekly mean male *Ae. aegypti* catches did not significantly differ between trap types. However, the rate of positive weekly detections of male *Ae. aegypti* was lower for the MAST Sticky than the other three trap types. The MASTs sampled significantly fewer mosquitoes other than male *Ae. aegypti*, than either the BGS trap or the SGAT. Also, the MASTs and SGATs all caught significantly less non-Culicidae bycatch than the BGS traps. Consequently, we have developed a versatile male *Ae. aegypti* trap which is potentially of great benefit to *Ae. aegypti* surveillance programs.

*Aedes aegypti* mosquitoes are competent vectors of the viruses causing dengue, Zika, yellow fever, and chikungunya (Paupy et al. 2009, Powell 2018). As their global distributions are projected to expand (Kraemer et al. 2019), these mosquitoes are of increasing concern to communities and public health staff worldwide. There still remains a large knowledge gap regarding dengue transmission and as such greater efforts in adult mosquito surveillance are recommended, including the evaluation of new trapping methods (Bowman et al. 2014).

Recently, there has been an interest in mosquito trap development to capture male *Aedes* mosquitoes (Johnson and Ritchie 2015, Balestrino et al. 2016, Pantoja-Sánchez et al. 2019). Male *Aedes* mosquitoes are being utilized in rear and release programs to control *Ae. aegypti* and *Aedes albopictus* (Skuse) (Diptera: Culicidae), so efficient monitoring of male densities is of interest (Carvalho et al. 2015, Ross et al. 2019). Additionally, while males do not transmit diseases and therefore have not been of primary interest to public health staff, they are a fundamental component of every established population of mosquitoes (Akaratovic et al. 2017) and are therefore indicative of the presence of populations within that location or the ability of a species to invade a port or cross a border. Furthermore, as males generally emerge 24–48 h before females they may also be indicative of future female activity within an area (Farajollahi et al. 2009). While there are other traps available that catch both female and male *Aedes* mosquitoes, there are a range of potential benefits to also deploying male-specific trap systems.

Currently, the BG-Sentinel (BGS) trap (Biogents, Regensburg, Germany) is one of the most commonly used adult *Aedes* surveillance traps. It is arguably the current gold standard, being more sensitive to detecting *Aedes* populations than other traps on the market (Kröckel et al. 2006, Williams et al. 2007, Gibson-Corrado et al. 2017). The BGS trap utilizes dark colors, which are known to attract *Ae. aegypti* (Christophers 1960, Ball and Ritchie 2010). Due to the effectiveness of the BGS trap’s visually attractive characteristics, this trap is routinely deployed without any additional chemical lures during surveillance activities run by organizations such as World Mosquito Program (globally) as well as Debug Innisfail and Queensland Health in Australia (Ritchie et al. 2013, Staunton et al. 2019, Ryan et al. 2020, Tantowijoyo et al. 2020). However, this trap is also commonly deployed with additional chemical lures. Williams et al. (2006) found that catch rates of *Ae. aegypti* in Australian field trials were not increased when BGS traps were baited with a BG blend kairomone lure, whereas Barrera et al. (2013) found significant increases in *Ae. aegypti* catch rates when they compared unbaited BGS traps to those baited with BG-Lures in cage trials in Puerto Rico as did Visser et al. (2020), when they added MB5 lures to BGS traps in field trials in Suriname. Additionally, baiting BGS traps with CO_2_ has been demonstrated to significantly increase catch rates of *Ae. aegypti* relative to traps baited with BG-Lures, in Florida (Wilke et al. 2019).

An additional reason the BGS trap can be so effective at catching mosquitoes is that it uses a fan to suck invertebrates attracted to its entrance funnel into a catch bag. Unfortunately, continually operating the BGS trap’s fan not only requires mains power or a 12-volt battery, but also results in catching a vast array of unintentional bycatch. Sorting this bycatch from the mosquito species of interest can be a labor-intensive process. Additionally, the fan desiccates and disrupts the specimens and therefore the trap must be serviced regularly to avoid degradation in specimen quality which complicates identification. Finally, the BGS trap is expensive (Johnson et al. 2018a), limiting its ability to be deployed in mass trapping programs or projects which have limited funds (Akaratovic et al. 2017). Consequently, there is a need for the development of an *Aedes* trap which requires limited electrical power, catches minimal bycatch, and can be left in situ for extended periods.

The BG-Gravid *Aedes* Trap (GAT; Biogents) is designed to capture gravid *Aedes* females (Eiras et al. 2014). This trap is cheaper than the BGS trap and passive (does not require mains power) as it uses water-based organic infusion to entice adults into the trap. Recently, a sound lure, mimicking the wing-beat frequency of the female *Ae. aegypti*, was deployed inside the GAT to attract conspecific males (Johnson and Ritchie 2015, Johnson et al. 2018a, Rohde et al. 2019), hereafter referred to as a Sound GAT (SGAT). Field data show that SGATs capture less female *Ae. aegypti* than the BGS traps (~20%), but similar numbers of male *Ae. aegypti*, relative to unbaited BGS traps (Johnson et al. 2018a, Rohde et al. 2019). While shown to be an effective trap for male *Ae. aegypti*, the SGAT still needs to be serviced regularly to reduce specimen damage from fungal growth associated with the infused water inside the trap.

Recent modifications to the BGS trap include the incorporation of sensor equipment which can be used to not only detect mosquitoes caught in the trap, but also communicate this information to surveillance staff—see the BG-Counter and BG-Eye by Biogents (2019). As different species may preferentially respond to different wing-beat frequencies, deploying specifically tuned sound lures in traps may increase the selectivity of trap catches (Belton 1994). This selectivity may be reduced if the animals do not choose to enter the trap and are instead sucked in with an active fan-based system. Therefore, remote sensing equipment may be further enhanced with a passive trap system, which could significantly reduce bycatch and therefore increase the accuracy of identification of remotely sensed catches. Also, both the SGAT’s and the BGS trap’s entrance funnels are relatively large and face upwards; therefore, these traps need to be sheltered to avoid negative impacts from rain falling inside them. A trap with a sidewards facing small entrance may offer better protection to electrical equipment from rainfall. Additionally, such a trap entrance may also only require a small and highly energy-efficient sensor system, which could be relatively cheap with minimal power consumption, thereby increasing the time between service regimes.

Lastly, testing various killing agents is vital to the development of passive mosquito traps. While insecticides, such as pyrethroid surface sprays, have been deployed in both GATs and SGATs (Ritchie et al. 2014, Johnson et al. 2018a), increasing global resistance of mosquitoes to such chemicals (Hemingway and Ranson 2000, Vontas et al. 2012) has resulted in a range of environmentally friendly alternatives also being deployed (Heringer et al. 2016). Such methods include using sticky cards and spraying the inside of traps with canola oil (Eiras et al. 2018, Johnson et al. 2018b). These insecticide-free trap types are highly attractive alternatives, especially in locations where insecticide resistance is a concern, if their catch rates are comparable to those which use surface spray.

We developed the Male *Aedes* Sound Trap (MAST) to be a new tool in *Aedes* mosquito surveillance. We ensured it had low power requirements, could run in the field for extended periods of time, and incorporate an insecticide-free killing method. We then compared the MAST’s ability to capture male *Ae. aegypti* and reduce rates of bycatch with unbaited (without any sound or chemical lure) BGS traps (version 2) and SGATs.

## Methods

### Trap Description

**Fig. 1.**
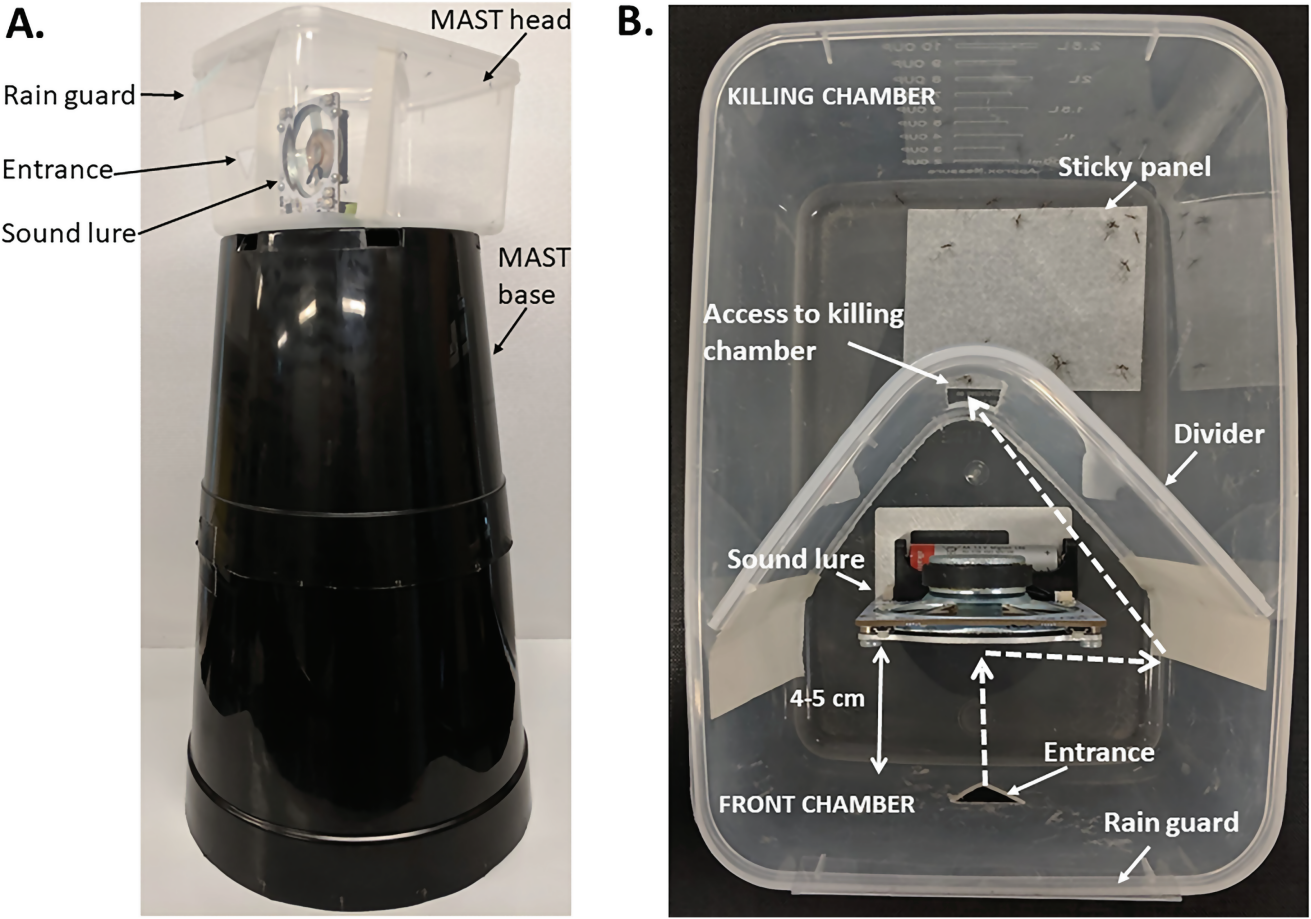
The MAST with (A) entire trap profile and (B) top view of the MAST Sticky head. Dotted arrows depict a potential path of the captured mosquito to the killing chamber.

The trap consists of two main components, a large black base and a clear head container which houses the sound lure and captures the mosquitoes (Fig. 1A). The black base consists of two potted-plant pots that stack to 45 cm (lower bucket dimensions of 230 mm diameter base, 285 mm diameter top, and 270 mm height with a carrying capacity of approximately 13.5 liters and upper bucket dimensions of 190 mm diameter base, 250 mm diameter top, and 230 mm height with an approximate carrying capacity of 7.5 liters; Garden City Plastics, Dandenong South, Australia). This large black base is a visual attractant for the male *Ae. aegypti* mosquitoes. Once the males are flying around the base they then hear the WBF tone played by a sound lure and enter the trap head.

The MAST head consists of a clear rectangular plastic container (210 mm long × 135 mm wide × 118 mm high, 2.5-liter; Kmart (Home and Co), Mulgrave, Australia) which, in its simplest form, only contains the sound lure. The trap entrance is a simple inverted equilateral triangle (2 cm in length) located in the middle of an end of the container. This type of MAST head could be sprayed with synthetic pyrethroid or oil to capture mosquitoes and, if desired, potentially contain a sensor system at the entrance to record insect entry.

The MAST head could also be divided into two chambers to create the MAST sticky, one housing the lure and the other housing a sticky panel (Fig. 1B). Once males fly into the head and contact the lure they then search for an exit. In their efforts to escape, they track along the walls and lid, flying into the corners of the structure. By placing a divider inside the container behind the sound lure we can direct the mosquitoes into a killing chamber away from the entrance. As they preferentially track toward corners they are less likely to exit out of the killing chamber once inside. This divider is made out of the corner of a plastic container from the same company, identical in materials to the 2.5-liter container, but is (290 mm long × 220 mm wide × 110 mm high, 5-liter; Kmart (Home and Co)).

Each divider is 18 cm long and 11 cm high. A rectangular section has been cut out of the middle of the divider (6 cm high and 1.5 cm wide, positioned 2 cm from the bottom) so that mosquitoes can access the killing chamber. The divider is taped (using 36 mm white masking tape, Bear, Saint-Gobain, Somerton, Australia) to the internal wall of the MAST 9 cm from the side with the entrance. In this position the divider corner is 6 cm from the back wall which leaves enough space for an 8 × 6 cm sticky panel to be laid flat on the MAST bottom in a corner of the killing chamber to fix the mosquitoes and prevent escape. Previous experimentation during trap development indicated that the sticky panel was best positioned on the container bottom rather than stuck to the container wall as it was easier to position and displayed higher catch rates.

While rain guards were not used in these trials as all traps were placed in sheltered locations, they have been developed for future trials aimed at sampling *Ae. albopictus*. Rain guards were cut from convention card holders (60 mm × 90 mm, Rexel, Shanghai, China). The side of the holder including the pin was simply cut off and, of the two remaining sides, the remaining short one (15 mm × 90 mm) was placed over the lip of the MAST container.

The sticky panels used were cards designed for the BG-Gravid *Aedes* Trap by Biogents. These cards were cut in half and the sections not glued were trimmed off so that each panel in the MAST was 8 × 6 cm.

The prototype version of the sound lure is designed and built by Verily and consisted of an 8-ohm 0.5-watt speaker mounted to a printed circuit board (PCB). The lure settings are highly adjustable and produce a sinusoidal tone of various frequencies with either an intermittent or continuous playback. The tone volume is also adjustable and the playback can be suspended in response to a photodetector determining low light, thereby extending battery life. During these field trials we used two similar lure prototypes. While both versions had the same speakers and tone settings, a revision of the PCB meant that the lures used in the first two trials were powered by two alkaline AA batteries while the lures used in the third trial required only one alkaline AA battery. This latest version of the sound lure is calculated to last up to 4 mo in the field. Batteries were replaced at the end of each trial. All lures set in MASTs produced tones of 500 Hz at 60 dB at trap entrance. Playback was intermittent at 30 s on-off and the photodetector was enabled so that the tone stopped playing between dusk and dawn. Identical lures were used in the SGATs with the same settings, except for the photodetector being disabled as the inside of the GAT was too dark to have similar operation to the MASTs. Subsequently, SGATs and BGS traps operated 24 h a day, whereas the MASTs only operated during the day.

### Field Trials

We ran three field trials over 12 wk using Latin square experimental designs within the Cairns city area, North Queensland, between 10 January and 9 May 2019. These trials compared catch results from MAST Spray and MAST Sticky versions, BGS traps, and SGATs. Traps were serviced and randomly rotated weekly. The BGS traps were unbaited version 2s and the SGATs contained a mild infusion (8 guinea pig food pellets/3 liters tap water; Ritchie et al. 2014) and the same sound lure as the MASTs. SGATs were only compared during two trials as an alternate MAST design aspect was tested instead during one trial due to developmental priorities.

### Data Analysis

Response variables investigated included the weekly capture rates of male *Ae. aegypti*, other mosquito bycatch (including female *Ae. aegypti*), all winged non-Culicidae bycatch, and all flightless bycatch. To analyze the influence of all trap types on catch rates we combined the two trials in which SGATs were deployed (*n* = 24). To include extra data, we also tested differences between BGS traps, MASTs, and MAST dividers by combining data from all three trials (*n* = 36).

All analyses were performed using the R statistical environment ver. 3.5.3 (R Core Team 2017). We fit the parameters ‘trap type’ and ‘square’ to each response variable’s count data by specifying a generalized linear model (GLM) with a negative binomial distribution (models with Poisson distributions were consistently overdispersed) and log link function using the *MASS* package (Venables and Ripley 2002). We then analyzed the effect of predictors with an analysis of deviance using the *car* package (Fox and Weisberg 2011). Finally, we performed post hoc Tukey comparisons to determine significant differences among the least-squares means of treatment groups using the *emmeans* package (Lenth et al. 2019). To model flightless bycatch we constructed a Bayesian GLM, using the *arm* package (Gelman et al. 2018), due to the perfect separation in data caused by the MAST consistently not catching any such invertebrates.

## Results

### Total Catches

We caught 987 mosquitoes in total throughout the Cairns region (Table 1). Of these, 460 were male *Ae. aegypti*, 105 in the BGS trap, 68 in the SGAT, 197 in the MAST Spray, and 90 in the MAST Sticky. The remaining 527 mosquitoes comprised of 267 female *Ae. aegypti*, 130 male and 112 female *Culex quinquefasciatus* (Say) (Diptera: Culicidae), 6 female *Aedes notoscriptus* (Skuse) (Diptera: Culicidae) as well as single females of: *Anopheles farauti* (Laveran) (Diptera: Culicidae), *Toxorhynchites* sp. (Theobald) (Diptera: Culicidae), and *Verrallina funerea* (Theobald) (Diptera: Culicidae). Additional taxa captured include various species of: Diptera (other than Culicidae), Lepidoptera, Collembola, Hymenoptera, Coleoptera, Araneae, Hemiptera, and Blattodea.

**Table 1.** Total invertebrate captures during field trial period for each trap type

Taxa	Winged	BGS	SGAT	MAST Spray	MAST Sticky
*Aedes aegypti* male	Yes	105	68	197	90
*Aedes aegypti* female	Yes	247	28	1	0
*Culex quinquefasciatus* male	Yes	121	3	3	3
*Culex quinquefasciatus* female	Yes	109	3	0	0
*Aedes notoscriptus* female	Yes	5	1	0	0
*Anopheles farauti*	Yes	1	0	0	0
*Toxorhynchites* sp. female	Yes	1	0	0	0
*Verrallina funerea* female	Yes	1	0	0	0
Diptera (other)	Yes	1,586	15	2	3
Lepidoptera	Yes	189	1	1	0
Collembola	No	56	0	0	115
Formicidae	No	22	0	0	7
Hymenoptera (other)	Yes	26	0	0	1
Coleoptera	Yes	21	0	0	0
Araneae	No	21	1	0	0
Hemiptera	Yes	12	0	0	0
Blattodea	No	1	0	0	0
Total		2,524	120	204	219

Note that SGATs were only run for two out of the three trials.

### Comparisons of Means for the Two Trials Including the SGAT

**Fig. 2.**
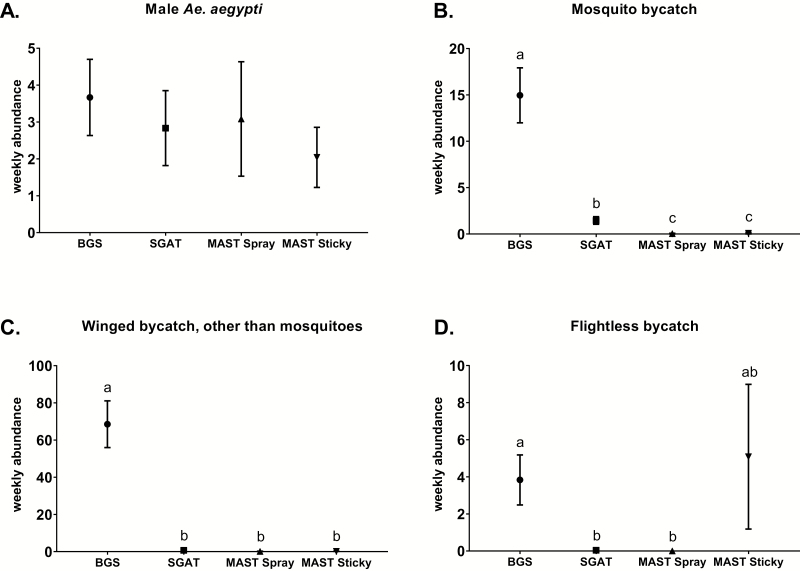
Trap capture weekly means (±SE) per trap type of (A) male *Ae. aegypti*, (B) mosquito bycatch, (C) winged bycatch other than mosquitoes, and (D) flightless bycatch from the two trials for each trap type, including SGATs. Different letters indicate significantly different groups (*P* < 0.05, Tukey HSD, *n* = 24).

No significant differences (⎕ ^2^ = 0.9, df = 3, *P* = 0.82, *n* = 24) were noted between mean weekly catch rates of male *Ae. aegypti* using BGS traps (3.7 ± 1; mean ± SE), SGATs (2.8 ± 1), MAST Spray (3.1 ± 1.6), and MAST Sticky versions (2 ± 0.8) deployed (Fig. 2A).

The BGS traps displayed significantly higher mean weekly catch rates of mosquito bycatch (15 ± 2.9) than the SGATs which displayed significantly higher mean weekly catch rates of mosquito bycatch (1.5 ± 0.4) than the MAST Spray (0.04 ± 0.04) and MAST Sticky versions (0.08 ± 0.08) deployed (χ ^2^ = 183.6, df = 3, *P* < 0.05, *n* = 24; Fig. 2B).

The BGS traps displayed significantly higher mean weekly catch rates of winged bycatch (68.5 ± 12.6) than the SGATs (0.7 ± 0.3), MAST Spray (0.13± 0.07), and MAST Sticky versions (0.13 ± 0.07) deployed (χ ^2^ = 356.3, df = 3, *P* < 0.05, *n* = 24; Fig. 2C).

Lastly, the BGS traps displayed significantly higher mean weekly catch rates of flightless invertebrate bycatch (3.8 ± 1.4) than the SGATs (0.04 ± 0.04) and MAST Spray (0 ± 0) with catches from the MAST Sticky versions (5.1 ± 3.9) not significantly different to other trap types deployed (χ ^2^ = 47.3, df = 3, *P* < 0.05, *n* = 24; Fig. 2D).

### Comparisons of Means for All Three Trials, Excluding the SGAT Data

**Fig. 3.**
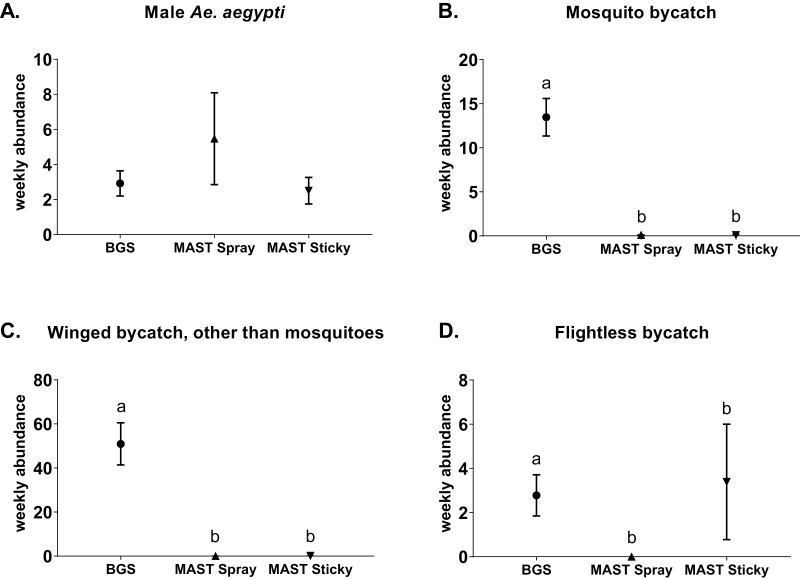
Trap capture weekly means (±SE) per trap type of (A) male *Ae. aegypti*, (B) mosquito bycatch, (C) winged bycatch other than mosquitoes, and (D) flightless bycatch from all three trials for each trap type. Different letters indicate significantly different groups (*P* < 0.05, Tukey HSD, *n* = 36).

There were no significant differences (χ ^2^ = 2.2, df = 2, *P* = 0.32, *n* = 36) between mean weekly trap capture rates of male *Ae. aegypti* between BGS traps (2.9 ± 0.7), MAST Spray (5.5 ± 2.6), and MAST Sticky versions (2.5 ± 0.8) deployed during all three trials combined (Fig. 3A).

The BGS traps displayed significantly higher mean weekly catch rates of mosquito bycatch (13.5 ± 2.1) than the MAST Spray (0.1 ± 0.05) and MAST Sticky versions (0.08 ± 0.06) deployed (χ ^2^ = 280.8, df = 2, *P* < 0.05, *n* = 36; Fig. 3B).

The BGS traps displayed significantly higher mean weekly catch rates of winged bycatch (50.9 ± 9.5) than the MAST Spray (0.08 ± 0.05) and MAST Sticky versions (0.1 ± 0.05) deployed (χ ^2^ = 505.9, df = 2, *P* < 0.05, *n* = 36; Fig. 3C).

Lastly, the BGS traps displayed significantly higher mean weekly catch rates of flightless invertebrate bycatch (2.8 ± 0.9) than the MAST Spray (0 ± 0) and MAST Sticky versions (3.4 ± 2.6) deployed (χ ^2^ = 22.5, df = 2, *P* < 0.05, *n* = 36; Fig. 3D).

BGS traps, MAST Spray and MAST Sticky versions positively detected male *Ae. aegypti* during 25 (69%), 26 (72%), and 20 (55%) wk, respectively, of the 36 weekly sampling periods for which they were run (Table 2). Additionally, male *Ae. aegypti* were positively detected in SGAT traps during 17 of the 24 wk (71%) this trap type was trialed.

**Table 2.** Positive detections of male *Ae. aegypti* during weekly sampling periods per trap type per trial

Trial	BGS trap	SGAT	MAST	MAST divider
One	9	9	9	8
Two	9	N/A	8	7
Three	7	8	9	5
Total (count)	25	17	26	20
Total (%)	69	71	72	55

Note that SGATs were only deployed 24 times unlike all other trap types which were deployed 36 times.

## Discussion

Both versions of MAST caught comparable weekly numbers of male *Ae. aegypti* to the BGS traps and the SGATs. These data suggest that male *Ae. aegypti* populations can be reliably estimated regardless of which trap is deployed throughout the northern Australian urban landscape. These findings are consistent with previous results where the catch rates of male *Ae. aegypti* were similar between SGATs and unbaited BGS traps (Johnson et al. 2018a, Rohde et al. 2019) and is a very promising outcome for a passive trap system such as the MAST.

The rates of weekly positive detections of male *Ae. aegypti* were also very similar between traps types, except for lower rates associated with the MAST Sticky version. This lower rate is suspected to be due to mosquitoes escaping the container, rather than being killed using a sticky panel. This version of the MAST is considered to be very important due to the increased rates of global insecticide resistance and the push for chemical-free mosquito traps (Heringer et al. 2016). Consequently, additional development is underway to improve the consistency of trap catches in this system.

Both versions of the MAST were very selective toward capturing male *Ae. aegypti* and caught significantly less other mosquitoes than either the SGATs or the BGS traps. The relatively reduced bycatch sampled in the MASTs is likely due to the sound lure being a selective attractant (Belton 1994), the lack of a fan indiscriminately sucking invertebrates into the BGS traps and the absence of any olfactory cue attractive the gravid females such as the SGAT infusion. The SGATs also caught less *Cx. quinquefasciatus* and female *Ae. aegypti* than the BGS traps. While gravid females are only a portion of the mosquitoes caught in BGS traps (Cilek et al. 2017), we also deliberately used a low amount of infusion (8 guinea pig food pellets per trap) in the SGATs to minimize the attraction of *Culex*—known to prefer larval habitats high in organic material (Day 2016)—and flies.

Finally, compared to the BGS traps, both MAST versions were highly selective against invertebrate bycatch other than mosquitoes. Most nonmosquito bycatch in the MAST traps can be attributed to a single event where a MAST Sticky caught a notable number of Collembola during a flood event. However, such captures are likely to be easily prevented with a simple application of petroleum jelly or a sticky barrier around the MAST entrance if desired.

This trap provides an attractive platform for an array of sensors which could detect and communicate male *Aedes* captures. The prototype was waterproof, modular, and stackable. This trap can be deployed after being sprayed by pyrethroids to kill captures or also as a chemical-free version with a sticky panel. The longevity of the sound lure and the fact that the MAST is a dry trap means that it can potentially last months in the field between services. Further testing is required to determine the efficacy of the MAST to capture male *Ae. albopictus*. Additionally, the influence of various sound lure frequencies, including frequency sweeps (Balestrino et al. 2016, Jakhete et al. 2017), on trap catches requires investigation.

The BGS traps in this study did not use lures as they are cheaper to deploy without chemical attractants, which makes them therefore of more interest to resource-constrained projects. Additionally, BGS traps are routinely deployed in mosquito control programs in Australia (Ritchie et al. 2013, Staunton et al. 2019) and by the World Mosquito Program in various developing countries including, but not limited to, when traps are deployed indoors and the odor is unwelcomed by occupants (S. A. Ritchie, personal communication; Tantowijoyo et al. 2020). A relatively early Australian study comparing female *Ae. aegypti* catches in unbaited BGS traps to those with a BG blend kairomone lure (consisting of lactic acid, caproic acid, and ammonia) found that such bait did not significantly change catch rates (Williams et al. 2006). Williams et al. (2006) compared the attractions of four different geographical strains of *Ae. aegypti* to chemical lures and suggested that the North Queensland (Australia) strain may have a relatively reduced attraction to kairomone lures in the absence of CO_2_ and that the visually attractive properties of the BGS trap may be more important than a kairomone lure when catching this species in this region. A later outdoor cage study in Puerto Rico demonstrated that BGS traps baited with BG-Lures (also containing lactic acid, caproic acid, and ammonia) caught approximately 1.5× more female *Ae. aegypti* than unbaited BGS traps (Barrera et al. 2013). Barrera et al. (2013) also found further significant improvements in catch rates of female *Ae. aegypti* when either CO_2_ alone or CO_2_ and BG-Lures were added to BGS traps baited with only BG-Lures. Wilke et al. (2019) demonstrated that BGS traps baited with CO_2_ (dry ice) caught over 2× more *Ae. aegypti* than those baited with BG-Lures. Lastly, Visser et al. (2020) compared unbaited BGS traps to those baited with MB5 (a blend consisting of five different volatile compounds including lactic acid and ammonia) and found that the baited BGS traps caught approximately 2× more female and 2.5× more male *Ae. aegypti* than unbaited traps. Clearly there is a wide range of chemical attractants that can be added to BGS traps with varying levels of influence on *Ae. aegypti* catches and future trials should compare catch rates of *Aedes* in MASTs and BGS traps with chemical attractants.

Lastly, our study aimed to compare the catch rates of male *Ae. aegypti* and bycatch in two MAST versions against those seen in two alternative trap systems. As such, we did not investigate the extent to which each trap estimates population sizes. Future research should also address this need and consider incorporating additional gravid traps or ovitraps as comparative trapping systems.

### Conclusion

We developed a prototype MAST that has low power requirements, includes a chemical-free option and is able to be left in situ for months. Both versions of MAST caught male *Ae. aegypti* as effectively as unbaited BGS traps and SGATs. Additionally, the MASTs were highly selective toward capturing male *Ae. aegypti* by catching significantly less other mosquitoes than both the BGS trap and SGAT and less nonmosquito bycatch than BGS traps. The MAST is therefore a potentially useful tool for sampling male *Ae. aegypti* in a variety of surveillance settings. With further development, these traps can potentially house sensor and communication equipment which may save considerable labor-related expenses with continued monitoring requirements during mosquito programs.
